# Exercise, Immune System, Nutrition, Respiratory and Cardiovascular Diseases during COVID-19: A Complex Combination

**DOI:** 10.3390/ijerph18030904

**Published:** 2021-01-21

**Authors:** Olga Scudiero, Barbara Lombardo, Mariarita Brancaccio, Cristina Mennitti, Arturo Cesaro, Fabio Fimiani, Luca Gentile, Elisabetta Moscarella, Federica Amodio, Annaluisa Ranieri, Felice Gragnano, Sonia Laneri, Cristina Mazzaccara, Pierpaolo Di Micco, Martina Caiazza, Giovanni D’Alicandro, Giuseppe Limongelli, Paolo Calabrò, Raffaela Pero, Giulia Frisso

**Affiliations:** 1 Department of Molecular Medicine and Medical Biotechnology, University of Naples Federico II, 80131 Naples, Italy; olga.scudiero@unina.it (O.S.); barbara.lombardo@unina.it (B.L.); cristinamennitti@libero.it (C.M.); cristina.mazzaccara@unina.it (C.M.); 2 Task Force on Microbiome Studies, University of Naples Federico II, 80100 Naples, Italy; 3 Ceinge Biotecnologie Avanzate S. C. a R. L., 80131 Naples, Italy; gentilelu@ceinge.unina.it (L.G.); ranieria@ceinge.unina.it (A.R.); 4 Department of Biology and Evolution of Marine Organisms, Stazione Zoologica Anton Dohrn, 80121 Naples, Italy; mariarita.brancaccio@szn.it; 5 Department of Translational Medical Sciences, Università degli Studi della Campania “Luigi Vanvitelli”, 80138 Napoli, Italy; arturocesaro@hotmail.it (A.C.); elisabetta.moscarella@unicampania.it (E.M.); amodio.federica@yahoo.it (F.A.); gragnano.f@gmail.com (F.G.); 6 Division of Clinical Cardiology, A.O.R.N. “Sant’Anna e San Sebastiano”, 81100 Caserta, Italy; 7 Unit of Inherited and Rare Cardiovascular Diseases, Azienda Ospedaliera di Rilievo Nazionale AORN Dei Colli, “V.Monaldi”, 80122 Naples, Italy; fimianifabio@hotmail.it; 8 Department of Pharmacy, University of Naples Federico II Via Montesano, 80131 Naples, Italy; slaneri@unina.it; 9 Department of Internal Medicine and Emergency Room, Ospedale Buon Consiglio Fatebenefratelli, 80123 Naples, Italy; pdimicco@libero.it; 10 Inherited and Rare Cardiovascular Diseases, Department of Translational Medical Sciences, University of Campania “Luigi Vanvitelli”, Monaldi Hospital, 81100 Naples, Italy; martina.caiazza@yahoo.it; 11 Department of Neuroscience and Rehabilitation, Center of Sports Medicine and Disability, AORN, Santobono-Pausillipon, 80122 Naples, Italy; ninodalicandro@libero.it; 12 Department of Cardio-Thoracic and Respiratory Sciences, Università degli Studi della Campania “Luigi Vanvitelli”, 80138 Napoli, Italy; limongelligiuseppe@libero.it

**Keywords:** coronavirus, physical exercise, immune system, nutrition, respiratory infection, cardiovascular disorders

## Abstract

Coronaviruses (CoVs) represent a large family of RNA viruses that can infect different living species, posing a global threat to human health. CoVs can evade the immune response, replicate within the host, and cause a rapid immune compromise culminating in severe acute respiratory syndrome. In humans, the immune system functions are influenced by physical activity, nutrition, and the absence of respiratory or cardiovascular diseases. This review provides an in-depth study between the interactions of the immune system and coronaviruses in the host to defend against CoVs disease.

## 1. Introduction

In recent years, new coronaviruses have emerged in various areas of the world, causing severe epidemics. Severe acute respiratory syndrome coronavirus (SARS-CoV) was first identified in Guangdong, China, in February 2003. The infection spread during the time period from November 2002 to July 2003, which infected around 8422 people worldwide, 916 of whom died.

Middle East respiratory syndrome coronavirus (MERS-CoV) was first identified in Jeddah, Saudi Arabia, in 2012 [1]. There were 1401 people affected, with 543 deaths [2,3,4,5]. Today, we are facing a new coronavirus: between the end of 2019 and the beginning of 2020, the Chinese city of Wuhan became the center of an outbreak of pneumonia of unknown cause. In January 2020, Chinese scientists isolated a new coronavirus, called Severe acute respiratory syndrome coronavirus 2 (SARS-CoV-2, known as 2019-nCoV), from affected patients [6]. Lastly, in February 2020, the virus was designated 2019 coronavirus disease (COVID-19) by the World Health Organization [7].

COVID-19 has reached humans through a spillover, a natural process whereby an animal pathogen evolves and becomes able to infect, reproduce, and transmit itself within the human species. This evolutionary phenomenon is found mainly in RNA viruses, such as coronaviruses when they have a higher mutation frequency than DNA viruses [8,9].

**Figure 1:**
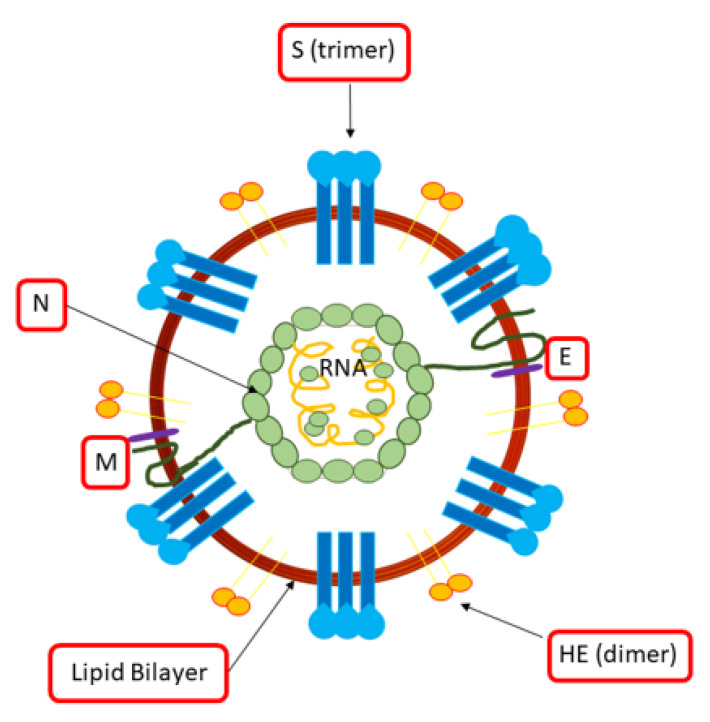
Morphological organization of the coronavirus.

Coronaviruses have a single-stranded linear RNA genome with the positive polarity of 27–32 Kb. They are spherical virions with a diameter between 80 and 220 nm formed by a phospholipid envelope, the pericapsid, which has distal projections called peplomers that give the virus a crown appearance (Figure 1). These viruses consist of different proteins: (i) protein N (50–60 kDa ) stabilizes RNA; (ii) glycoprotein S (80–220 kDa ) forms pleplomers, which favor the attack of the virus and fusion with the host’s cell membrane; (iii) glycoprotein M (20–30 kDa ) interacts with the nucleocapsid; (iv) protein E (9–12 kDa) is a constituent of the envelope and interacts with the glycoprotein M to stimulate budding; (v) hemagglutinin-HE (120–140 kDa ) is involved in the release of the virus (Figure 1).

From a genotypic and serological typing, CoVs are divided into four categories: α, β, ɣ, and δ. In addition, the β-CoVs are subdivided into four other lineages A, B, C, D. These viruses can cause a variety of respiratory conditions, ranging from the common cold to Middle Eastern respiratory syndrome (MERS) to severe respiratory syndrome (SARS) [2,3,4,5].

**Figure 2:**
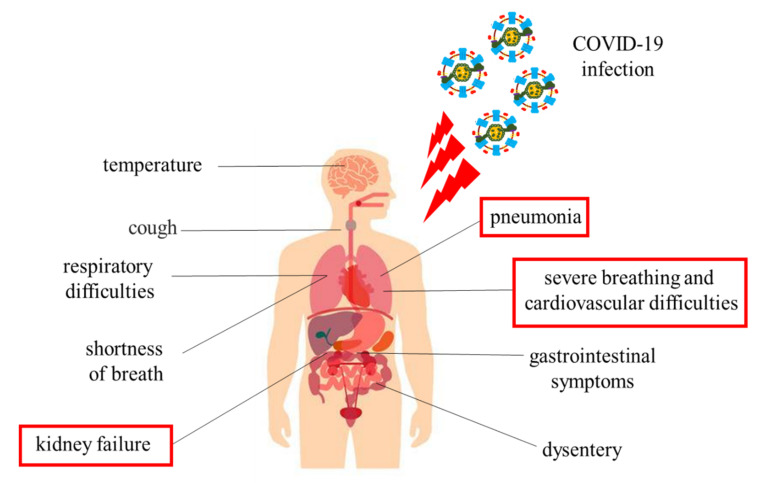
Symptoms of coronavirus.

The most common symptoms are fever, headache, cough, breathing difficulties, and diarrhea (Figure 2). In some cases, these symptoms may remain silent; on the other, the manifestation is violent to the point of causing severe pneumonia, dyspnea, renal failure, and even death (Figure 2) [10]. Patients most vulnerable to infections have pre-existing diseases, including diabetes, hypertension, cardiovascular disease, and chronic inflammation of the upper respiratory tract (URTI) [7,10].

During virus infections, the host activates the immune system to fight the pathogenic microorganism. An out of control immune response may occur during a violent infection such as COVID-19, resulting in substantial lung tissue damage [9,10].

In this scenario, several scientists wonder if the planned physical activity could disadvantage or favor the taking root of the pathology induced by COVID-19.

The purpose of his review is to shed light on how, during COVID-19 infections, the correct functioning of the human immune system can be influenced by intense exercise, nutrition, and pre-existing respiratory or cardiovascular diseases.

## 2. The Immune System

The immune system is a complex system of cells, tissues and organs with specific functions. Immune circulating molecules recognize and eliminate foreign agents: (bacteria, parasites, fungi, viruses, cells infected with pathogens and cancer cells). The immune system implements two forms of defense: innate immunity and or acquired immunity [11,12].

### 2.1. The Innate Immunity

Innate immunity consists of pre-existing mechanisms for meeting the foreign agent and acting against pathogens that are recognized as a threat. It has been present since birth, including both the body’s barriers that are circulating cells and proteins, which act as regulators and mediators of the body’s inflammatory response [12].

A key role is also played by the cellular component and the antimicrobial peptides of innate immunity [12,13,14].

Defensins, also called antimicrobial peptides (AMP), are proteins used to defend an organism from attack by pathogens [11,14,15,16,17,18,19,20,21]. They are a family of very ancient proteins highly conserved in mammals, insects and plants. There are two main categories of defensins: alpha and beta, differing in the type of producing cell and in their localization. Alpha-defensins are mainly produced by neutrophils and Paneth cells. Beta-defensins are produced by epithelial cells belonging to the respiratory tract, the integument, the urogenital tract and the tongue.

Human alpha-defensin 1 (HNP1) can inactivate herpes virus simplex type I and type II (HSV-1 and HSV-2) and cytomegalovirus (CMV) [22]; on the other hand, human beta-defensin 2 (HBD2) and human beta-defensins 3 (HBD3) bind directly to and inactive HSV-1 [23].

### 2.2. The Acquired Immunity

Acquired immunity develops instead after birth, during the first year of life, and is enhanced in response to infections and foreign agents’ presence. Being a response that the organism custom-made according to the foreign agent, adaptive immunity is much faster and more effective than innate immunity. The response mechanism of specific or adaptive immunity is made possible thanks to T and B lymphocytes [12].

MERS-CoV and SARS-CoV are β-coronaviruses, which can induce serious and lethal respiratory tract infections [24]. In this injury, the main role is played by T cells, CD4 + T cells and CD8 + T cells, which have a consistent antiviral activity against the pathogens. However, their activation, if disproportionate, can cause autoimmunity and consequently lead to a malfunction of the immune system exacerbating the pathology. During CoVs infection, CD4 + T cells promote the production of virus-specific antibodies by activating T-dependent B cells; while, CD8 + T cells are cytotoxic and can therefore kill cells infected by the viral agent. After activation of the CD4 + and CD8 + T cells, the helper T cells are triggered. These cells produce pro-inflammatory cytokines, including interleukin-17 (IL-17) using the kappa-light-chain-enhancer nuclear-activated signaling pathway of activated B cells (NF-kB) [25]. The activation of these cytokines allows the recruitment of monocytes and neutrophils at the infection site, which in turn activated other cytokines such as IL-1, IL-6, IL-8, IL-21, TNF–β [25], giving rise to the cascade cytokines, which is one of the main causes of respiratory complications caused by CoVs.

### 2.3. Mechanism of Immune Systems in the Human Body against COVID-19

Recently, *Lucas* et al. analyzed blood samples taken over time from individuals hospitalized with moderate or severe COVID-19 [26]. Such information is useful for efforts to try to predict the individuals at risk of developing a severe form of the disease, which is often accompanied by an intense immune response. The authors identified a subset of immune-signaling molecules called cytokines that are expressed in people with moderate or severe disease; IFN-α is one such “core” cytokine. The expression level of certain other cytokines, such as IFN-λ, mainly changed when the disease became more severe. In addition, some inflammation-promoting cytokines, such as TNF-α, correlated with viral load in the nasal passages. Moreover, viral load declined over time in people with moderate COVID-19, but not in those with severe disease. Finally, the levels of CD4 and CD8 T cells, which are key immune cells involved in viral clearance, were lower in people with moderate or severe disease than in healthy individuals uninfected with SARS-CoV-2, the virus that causes COVID-19 [26]. This study underlines that the molecular mechanisms underlying the activation of the immune system due to COVID-19 are complex and that further studies will be needed to have a clear overview.

## 3. Effect of Exercise on Immunity: Open Window Theory

**Figure 3:**
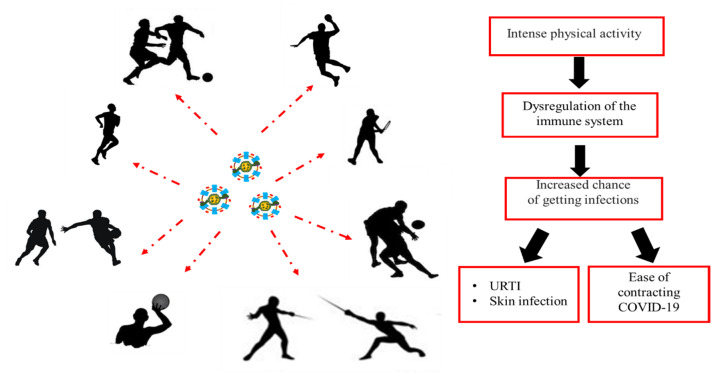
The immune system of athletes.

Most studies on the effects of exercise on the immune system have been carried out [27,28,29,30] by evaluating some parameters before and after physical performance. Single bouts of moderate-intensity exercise are “immunoenhancing”, and in fact, are responsible for a reduction in inflammation, maintenance of thymic mass and enhanced immunosurveillance [31]. On the other hand, some studies show negative changes in the levels and function of many components of the immune system in response to intense and prolonged exercise. During this phase, called the “open window”, the host is more sensitive to microorganisms such as viruses and bacteria with a greater risk of contracting infections (Figure 3) [32]. Different mechanisms contribute to these alterations, such as stress resulting from intense exercise, changes in the concentration of hormones, cytokines and in body temperature, increased blood flow, lymphocytic apoptosis and dehydration.

In particular, high-intensity endurance exercise has been linked to a change in the white blood cell count of athletes, such as an increase in granulocytes and monocytes, a decrease in lymphocytes and an increase in neutrophils and eosinophils [33].

An interesting role seems to be played by “natural killer” cells, which activity seems exalted during physical effort, with an increase in CD16 + cells [34].

Moreover, after intense physical exercise, there is lower immune protection of the upper airways due to a decrease in nasal and salivary secretions with low IgA levels, an increase in the nasal ciliary mucus transit and a compromised nasal function of neutrophils [35,36,37].

Based on this knowledge, athletes could be exposed to a greater risk of contracting COVID-19.

Moreover, professional athletes have a greater susceptibility to chronic inflammations of the upper respiratory tract (URTI) and to pathologies of the skin tissue (Figure 3) [11,38].

Pathogenic microorganisms such as *Streptococcus pneumonia, Hemophilus influenza, Moraxella catarrhalis*, *Streptococcus pyogenes and Staphylococcus aureus* can cause URTI [11,38]. The susceptibility to URTI after physical exercise has been described with a J-shaped curve [39]. According to this model, moderate activities reduce the risk of URTI compared to sedentary subjects. In contrast, strenuous exercise is associated with an increased risk of URTI.

The J-curve model cannot be applied to elite athletes [40]. In fact, a high training load in these athletes is associated with a lower risk of infections [41]. Thus, the J-curve model takes an S-shape when elite athletes are included [41]. The S-curve model describes a relationship between exercise workload and risk of infections: low and high exercise loads increase the infection odds ratio, while moderate and elite exercise loads decrease it.

At the same time, professional athletes are affected by skin infections, often caused by *Staphylococcus aureus* [11,38]. Furthermore, the intended sport can cause an alert of the immune system determining a momentary immune compromise of the athlete who will be more predisposed to the infection diseases (Figure 3) [11,38]. Moreover, contact sports have a greater risk in the appearance of infections caused by the sharing of environments and equipment and by physical contact [11,38]. Following these, since the appearance of the COVID-19 pathology, it was decided to suspend all competitive activities and training to avoid the appearance of outbreaks within sports clubs since the contagion increases with physical contact and takes root with greater violence in subjects with a compromised immune system.

## 4. Nutrition and Prevention

**Figure 4:**
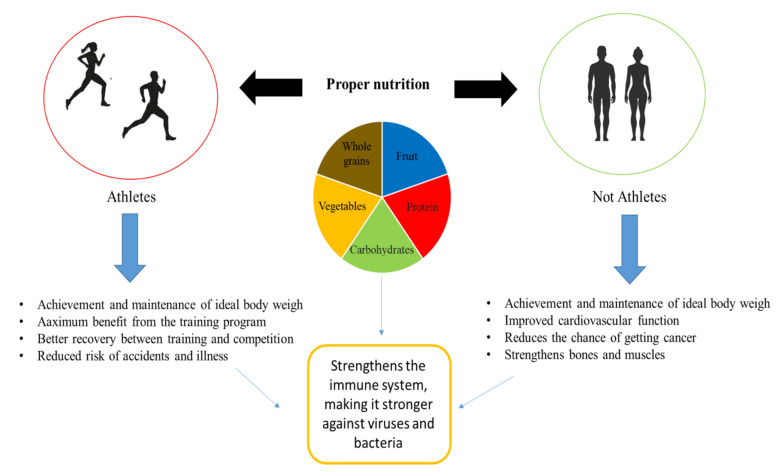
Proper nutrition.

Nutrition plays an essential role in the development and maintenance of the immune system. Nutritional deficiencies can compromise the immune response and increase susceptibility to infections. Instead, a good nutritional status can prevent the development of diseases and immune depression (Figure 4).

Resistance to infections can be improved by providing the body with antioxidants. Antioxidants are molecules that help defend against the attack of harmful agents and the state of oxidative stress. In fact, they are able to prevent or repair damage caused by free radicals. The most powerful antioxidant in the human body is glutathione. Several studies have recently been reported on how natural and edible molecules can exert an antioxidative activity and protect against inflammatory diseases by interacting with the enzymes involved in the synthesis of glutathione [42,43,44]. Guloyan et al. also demonstrated that glutathione (GSH) supplementation as adjunctive therapy in COVID-19 [45]. Specifically, if GSH levels decrease, there is a simultaneous increase in pro-inflammatory factors such as interleukin-6 (IL-6) and tumor necrosis factor-α (TNF-α), and vice-versa: an increase in GSH levels inhibits these factors and helps the cell to survive. Consequently, it would favor the patient’s recovery [45].

At the same time, vitamins/pro-vitamins are essential. In fact, vitamins play a fundamental action in the regulation of many chemical reactions that take place in our body and which are essential for our life. Specifically, they help to supply energy to the body and ensure cell renewal, preventing the onset of some diseases such as neurocognitive diseases, muscle damage and cardiac disorders [46,47].

Recent studies have highlighted a key role of vitamin D in COVID-19-positive patients. On one hand, vitamin D appears to be a negative endocrine renin–angiotensin system (RAS) modulator; on the other hand, vitamin D increases the expression and concentration of ACE2, MasR and Ang- (1–7) and has a potential protective role against acute lung injury/acute respiratory distress syndrome [48].

Vitamin C also appears to play a pivotal role in COVID-19-positive patients [49]. In particular, patients with acute respiratory infections such as pneumonia or tuberculosis have reduced plasma concentrations of vitamin C; however, administration of vitamin C has been shown to reduce the severity and duration of pneumonia in elderly patients [50].

In COVID-19 patients, a vitamin C supplement has been shown to decrease the increase in pro-inflammatory cytokines such as IL-6 and TNF-α, and at the same time, stimulate the production of anti-inflammatory cytokines such as interleukin -10 [51]

Other micronutrients useful to keep the immune system efficient and ready to react to external aggressions are vitamin E and selenium. Vitamin E and selenium both act through antioxidant pathways to increase the number of T cells, enhance mitogenic lymphocyte responses, increase IL-2 cytokine secretion, enhances NK cell activity, and decrease the risk of infection [49].

Finally, an essential role in COVID-19 patients is also played by vitamin A and magnesium [49]. A deficiency of vitamin A and magnesium is associated with an increase in IL-6, a key pro-inflammatory factor in acute respiratory syndrome; therefore, a correct intake of these micronutrients blocks this increase, favoring a patient’s recovery [49].

Strong immune defenses also pass through a healthy intestine. For the health of the intestinal microbiota is necessary to regularly take probiotic foods [15,16,52,53,54].

Proper nutrition is not only necessary for COVID-19 patients but also for those who are preparing to carry out post-COVID-19 motor rehabilitation and for those who have been forced to lockdown. In particular, the San Donato group of the San Raffaele Hospital has drawn up a “booklet” which describes both the diet and the physical activity to be performed in order to combat this period of pandemic (www.grupposandonato.it).

Therefore, proper nutrition and a healthy lifestyle accompanied by moderate exercise (Figure 4) can be supportive for those who have fallen ill with COVID-19, but at the same time, they are necessary for those living in confinement in order to increase risk factors such as obesity and cardiovascular disorders [55,56].

## 5. Respiratory Infection and Exercise: Risk and Complications of COVID-19

Competitive athletes often present respiratory tract infections caused by both viral agents, such as *Rhinovirus*, *Adenovirus* and *Coronavirus*, both bacteria, such as *Streptococci* and *Staphylococci*. Such infectious processes are called upper respiratory tract infection (URTI) [57]. Hence, it is necessary to pay attention to the spread of COVID-19 within the sports community.

Respiratory symptoms dominate the clinical manifestations of COVID-19, and some patients show severe cardiovascular damage [58]. COVID-19 primarily affects the respiratory system causing pneumonia and acute respiratory distress syndrome (ARDS) [58,59,60,61]. In the initial phase of COVID-19 infection, patients present with typical flu symptoms such as cough, fever, respiratory fatigue, which then result in acute pneumonia. In the later stages, both intra- and extra-respiratory changes are evident [49,50,51]. The progression of these symptoms is extremely rapid [58,59,60,61]. Respiratory pathological findings of COVID-19 in the early-stages are rare; however, Tian and co-authors [60] reported that infected patients could show proteinaceous exudate, edema, focal reactive hyperplasia of pneumocytes with sporadic inflammatory cell infiltration and multi-nucleated giant cells. In addition, reactive alveolar epithelial hyperplasia and fibroblastic proliferation have been reported in some patients. These manifestations are caused by the ability of the virus to escape the immune system; the latter, consequently, once activated, generates a cascade of cytokines that affect the respiratory component.

In athletes, intense exercise induces a systemic response, leading to the activation of the immune system [61,62,63]. Uncontrolled activation of the immune system can increase the risk of infection or induce inflammatory processes in the airways [61,62,63].

Moreover, elite athletes sensitive to URTI show an altered inflammatory response [64], and in some cases, this alteration can be caused by a genetic predisposition [65,66,67]. Therefore, athletes also have some immune limits, and their intrinsic genetic profile, in combination with other stressors and environmental factors, will determine their risk profile for URTI.

However, specific data regarding the prevalence, nature and behavior of COVID-19-related disease in athletic individuals are currently not available.

Thus, although the data collected to date indicate that young athletes are less likely to die from COVID-19, it is still necessary to continuously monitor both the athletes and the areas used for sports in order to decrease the infection and the spread of the virus.

## 6. Cardiovascular Involvement in COVID-19: Direct Injury and Impact of Cardiac Comorbidities

COVID-19 has been observed to lead to cardiac manifestations such as arrhythmias, myocardial injury and heart failure. Patients with previous cardiovascular comorbid conditions have an elevated mortality rate [68,69].

The association between cardiovascular diseases and COVID-19 is affected by potential limitations due to several factors, such as age and pre-existing cardiovascular disease; the latter seems to be linked with worse outcomes and increased risk of death in patients with COVID- 19 [70]. Different countries and different areas of the same country have adopted different strategies both in terms of hospitalization and test for COVID-19 [71]: these factors may be responsible for selection bias and may have influenced the estimates of the virus impact in terms of the characteristics of the examined populations and deaths.

Cardiac involvement in COVID-19 infection can have 2 expressions: (i) direct injury to the heart; (ii) effects of cardiac comorbidities on the prognosis of infection. Based on information from the Chinese National Health Commission, many people, who were later diagnosed with COVID-19, had initially contacted their doctor for cardiovascular symptoms, in particular palpitations and chest pain [6].

In a study conducted in China on 41 patients with COVID-19, 5 of them showed an increase in high-sensitivity cardiac troponin (>28 pg/mL) [58]. In another study conducted on 138 patients, 36 had severe symptoms and were treated in intensive care [72]. Among the latter, an increase of cardiac markers of necrosis was reported (median creatin-kinase concentration of 18 U/I compared to 14 U/I). These data suggest that patients with severe symptoms can have complications affecting myocardial tissue.

In patients with COVID-19, the incidence of cardiac manifestations may be high. The intense inflammatory response and hemodynamic changes occurring during severe forms of the disease could contribute to the rupture of atherosclerotic plaques resulting in acute coronary syndromes (type I myocardial infarction) [73]. The inflammatory cells can produce cytokines such as interleukin-1 and interleukin -6 and tumor necrosis factor, mediators that can enter the systemic circulation. Systemic cytokines storm in COVID-19 could stimulate leukocyte adhesion molecule expression, increasing inflammatory cells in atherosclerotic lesions and leading to plaque rupture [74]. This local response to systemic stimuli is defined as the “echo” phenomenon [75]. In addition, hypoxemia, especially in the context of severe infections and ARDS, is an element potentially responsible for a mismatch between supply and demand with the development of type II myocardial infarction. In addition, abnormalities of the electrocardiogram and echocardiogram can be detected with high-frequency in these patients. These alterations have also been related to a serious manifestation of disease and worst prognosis.

Several cases of acute myocarditis related to COVID-19 have been described, and it was hypothesized that myocarditis might have contributed to the clinical worsening of the patients [72].

However, on the basis of the data concerning the prognostic value of troponin in hospitalized patients with COVID-19 [75,76], a troponin dosage should be performed, as a prognostic indicator, in all patients with COVID-19 at admission, periodically during hospitalization and in case of clinical deterioration.

Furthermore, COVID-19 seems to be responsible for both a worsening of pre-existing heart failure and the manifestation of de novo heart failure [6].

Arrhythmias have been described in several cases of patients with COVID-19: the palpitations were present as presenting symptoms in patients admitted to hospital with a diagnosis of COVID-19 [61], and cardiac arrhythmias were more frequent in patients admitted to intensive care [77].

A meta-analysis of patients with COVID-19 reported a prevalence of hypertension, cardio- and cerebrovascular disease and diabetes [78]. Patients who required hospitalization in intensive care were more frequently affected by comorbidities.

Elite athletes, being young and potentially without cardiovascular disease, could be exposed to cardiological involvement due to direct damage from COVID-19.

There are multiple mechanisms of cardiac injury in patients with COVID-19, and one of the most common is thrombotic diathesis. In fact, these patients are in a procoagulant state due to an increased level of tissue factor and fibrinogen influenced by impaired fibrinolysis [74]. For these reasons, athletes are not risk-free for thrombotic complications in COVID-19 infection, having an increased basic thrombophilic risk [79].

All major sports leagues have been suspended, annulled or postponed due to COVID-19 since early March 2020 because training sessions and sporting events can be one of the major vehicles for the spread of the infection [6]. In particular, athletes who practice team sports are more prone to contact, and some cases of infection have manifested first in a single athlete and then in other teammates [80]. COVID-19 infections have been reported among competitive athletes, but these have had a favorable outcome, and there are currently no data of cardiac involvement in these patients’ settings.

To date, the sequelae that COVID-19 infection can cause are unclear, and the long-term results of cardiac involvement in recovered patients are not known. For these reasons, based on the “open window” theory, a conservative procedure would be to recommend athletes to control training sessions, and prevention of COVID-19 is essential for the competitive athlete to minimize the adverse effects that could happen on his or her respiratory tract, cardiovascular system, and aerobic ability in both the short and long-term period.

## 7. The Association between Sport and Venous Thromboembolism

Prolonged exercise induces hemoconcentration because of strong sweating and dehydration [80,81,82]; furthermore, the prolonged activity of muscles may induce vascular compression and vascular wall damages, leading to endothelial dysfunction in particular for women [83,84] that induces a hypercoagulable state [85]. In particular, an acquired resistance to protein C has already been underlined [86].

To these specific acquired conditions induced by the exercise per se, we should add that there always known or unknown personal trend to thrombosis in each subject [79]; everyone, in fact, may be an asymptomatic carrier of prothrombotic conditions as inherited or acquired thrombophilia and in addition, may take pharmacological treatments at risk for thrombotic events as oral contraceptive use [87].

On the other hand, a particular predisposition to develop a severe clinical form of SARS during the COVID-19 outbreak has been identified for people that is runner or that performs frequent exercises [88]. Actually, there are no specific mechanisms that may be associated with this trend, but several pathophysiological ways may be hypothesized. First of all, physical activity may have effects on physiological hormone production with a trend to hyperproduction of androgenic hormones [89]; the hyperproduction of androgenic hormones is associated with several effects that lead to a hypercoagulable state [90]: an increase in cholesterol and other atherothrombotic lipoproteins, decreased fibrinolytic actions and reduction of clotting inhibitors as antithrombin and protein S has been reported.

## 8. Genetic Predisposition to COVID-19 Infection and to Athlete Performance

There is the first evidence that a predisposing genetic background may contribute to interindividual disease susceptibility and/or severity. In fact, polymorphic variants in *ACE2* and *TMPRSS2* genes may be responsible for different host responses to COVID-19 infection [91,92,93]. These genes encode the host entry receptor (ACE2) and the cellular serine protease 2 (TMPRSS2), a host cell protease, essential for viral spike protein (glycoprotein S) priming.

Clinical manifestations of COVID-19 infection seem also related to a polymorphic variant in the *ACE1* gene, the gene encoding the angiotensin-converting enzyme 1, which is a close relative of ACE2 in blood pressure control. The deletion/insertion polymorphism (D/I) in intron 16 of *ACE1* gene is associated with both modifications in circulating and tissue concentrations of ACE enzyme, both with the expression level of ACE2, deletion variant being associated with reduced ACE2 expression level [94].

Using data from 33 countries (spanning a region from Portugal to Moldova and from Saudi Arabia to Finland), the researchers showed that the variability in COVID-19 prevalence is negatively correlated with the ACE D allele frequency [95,96].

Genetic susceptibility to COVID-19 may be, also, linked to the genes encoding human leukocyte antigens (HLAs) or the Toll-like receptors (TLRs). Nguyen et al. found an in silico link between specific HLA genes and the severity of COVID-19. Particularly, the HLA-B*46:01 and the HLA-B*15:03 variants show the fewest and the greatest predicted binding peptides for COVID-19, respectively. Therefore, patients carrying the first variant may be particularly susceptible to COVID-19; in contrast, patients carrying the HLA-B*15:03 variant may have some protection [97]. Furthermore, in silico analysis highlighted that the COVID-19 genome contains a large number of ssRNA fragments that could be intercepted by host Toll-like receptors 7 and 8 (TLR7/8), promoting a robust pro-inflammatory response [98].

Finally, a correlation was also found between the ABO group and COVID-19 susceptibility, as already described for other coronavirus infections. Blood type A appears to be associated with a higher risk of contracting the COVID-19, whereas type 0 offers the best protection. It may be related to the inhibition of adhesion of COVID-19 protein to ACE2-receptor caused by anti-A antibodies [99].

Genetic background is an important step in determining athletic performance, such as strength, power, endurance, and other phenotypes, contributing to a predisposition to success in certain types of sport. Actually, 66% of phenotypic variability in athlete performance may be explained by genetic factors [100]. At least 155 genetic markers are associated with elite athlete status [101]. Of note, some genetic markers associated with athletic performance have recently been associated with COVID-19 infection. ACE D/I is among the most studied polymorphisms related to the variability of physical performance characteristics in response to endurance or strength training. Several studies have associated the ACE I variant to endurance performance; conversely, the ACE D polymorphism is associated with an increased percentage of fast-twitch muscle fibers and increased isometric and isokinetic muscle strength [101].

In addition, regular physical exercise enhances resistance to many infections via the TLR signaling pathways [102]. Both acute aerobic and chronic resistance exercise causes the decreased expression of TLRs, reducing inflammatory cytokine production. This effect contributes to post-exercise immune-depression; however, over the long term, it can play a beneficial role because it decreases the whole-body chronic inflammation [103].

Finally, athletes with O blood groups have better performance in endurance running, highlighting the influence of the ABO blood group on sports performance [104].

## 9. The State of Art about Sport and COVID-19

The measures introduced by the government to limit the spread of the virus have also hit the sports world. Starting from March 2020, all competitive and noncompetitive sports have been suspended. A drastic, but necessary measure, in fact, avoiding personal contacts and practicing intense personal hygiene are the main tools to avoid contagion., but it is clear that in some sports, particularly contact sports such as football and basketball, avoiding physical contact is almost impossible [105]. The only possible workouts were individual ones, indoors or outdoors.

The world of sport has been turned upside down; not only can athletes no longer train and compete, but even important events such as the Tokyo Olympic Games (OGs), the Euro (European Championships in Association Football), and the Wimbledon tennis tournament scheduled for 2020 have been delayed [106].

Numerous studies have evaluated the effects of restrictions imposed on athletes. It emerged that the lockdown period had a negative impact on the athlete’s health [107]. Pillay et al. have reported that many athletes felt depressed (52%) and have struggled to keep themselves motivated to train (55%) [108]. Moreover, the habits of athletes have been changed: 76% of them, in fact, have consumed excessive amounts of carbohydrates during the quarantine period.

**Figure 5:**
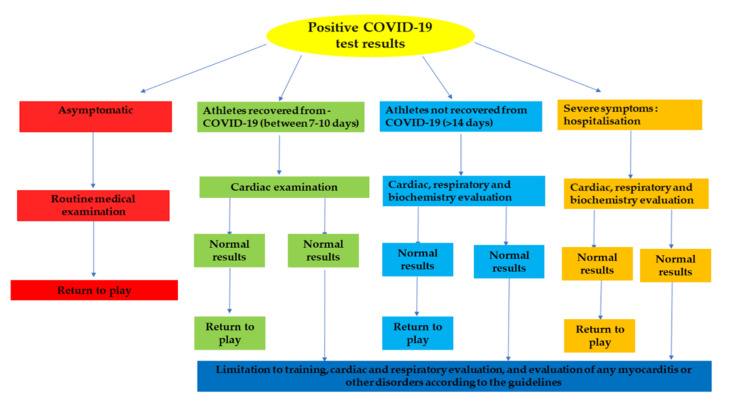
Protocols for return-to-play for the athletes.

There are numerous questions regarding the strategy to be applied for the athletes to return to play. Since the majority of the population, athletic and otherwise [109,110], developed myocarditis from SARS-CoV-2, and athletes with mild a moderate COVID-19 infection are reporting a persistent cough and dyspnea following infection, especially in the context of strenuous physical activity, it was considered appropriate to subject athletes to a series of diagnostic tests before returning to play. The type of protocol to follow varies according to the severity of the COVID-19 infection, see Figure 5.

However, in May 2020, sports competitions restarted, but in October 2020, in consideration of the particularly widespread nature of the virus and the increase in cases on the national territory, limitations were again introduced. Since then, only sports events and competitions organized by international sports organizations have been allowed behind closed doors, that is, without the presence of the public. Furthermore, activities in gyms, swimming pools, swimming and ski centers have been suspended.

## 10. Physical and Mental Rehabilitation through Moderate Exercise

Although COVID-19 disease occurs mainly with full-blown breathing problems, there are other symptoms that emerge in the post-acute phase. In this phase, patients can be physically and emotionally debilitated [111], showing signs of asthenia, difficulty in movement, a deficit of the peripheral and central nervous system, such as loss of smell and taste. Hence, it is necessary to create a multidisciplinary team of nurses and specialist doctors. At present, there are no shared guidelines for respiratory physiotherapy aimed at patients affected by COVID-19. Intensity and duration of rehabilitation depend, in general, on how long the hospital stay has been. COVID-19 is a disease that severely tests the strength and efficiency of the respiratory muscles. For those who have just returned from an intensive care unit stay, a course of at least 2–3 weeks may be necessary. However, a period of between 5 and 10 days is almost sufficient for all other patients.

Pending the guidelines, a group of Italian experts has published a document entitled: “Managing the respiratory care of patients with COVID-19: Italian recommendations, European Respiratory Society” [112]. In a vade mecum published on the website of the European Respiratory Society [113,114], extenders still refer to the need for personalized treatments taking into account the probable loss of weight and muscle mass [115,116]. To those who before the COVID-19 infection already lived with hypertension, arrhythmia, heart failure or had suffered a heart attack (in the last three months), experts advise against any activity without having first received the evaluation of a specialist (cardiologist, pulmonologist, sports doctor) and in the absence of a physiotherapist [115]. The rehabilitation program planned for COVID-19 patients has the purpose of improving respiratory dynamics, counteract musculoskeletal problems, and rehabilitate the person from a neuropsychological point of view.

COVID-19 patients usually have higher levels of anxiety and depression [117], which can also affect the patient’s immune system [118]. Stress can alter immunity by compromising the balance of defense cells; in this case, it alters the balance between T-helper-1/T helper-2. Stress can cause this alteration through its effects on increasing the levels of serum corticosteroids and catecholamine hormones. Therefore, a decrease in the immune response may occur. The aerobic exercise that physiotherapists undergo post COVID-19 patients can cause a decrease in serum corticosteroids and catecholamine hormones, restoring the proper functioning of the immune system by restoring the T-helper-1/T-helper-2 relationship [116]. Furthermore, cycling for 15 minutes has a positive effect on both young and old people with anxiety disorders [119]. Moreover, walking with 50% of maximum heart rate or running with 60–90% of maximum heart rate for 20 minutes significantly reduces sensitivity to anxiety. In addition, aerobic exercises for just three days significantly reduce emotional arousal to any unpleasant stimuli. Finally, regular mild aerobic exercises significantly reduce anxiety disorders [120].

In general, moderate exercise accompanied by the supervision of specialists can improve the general health and at the same time allow the recovery of a normal lifestyle where the correct functioning of the motor, respiratory and mental units has been undermined.

## 11. Diagnostic Methods for Detecting COVID-19

**Figure 6:**
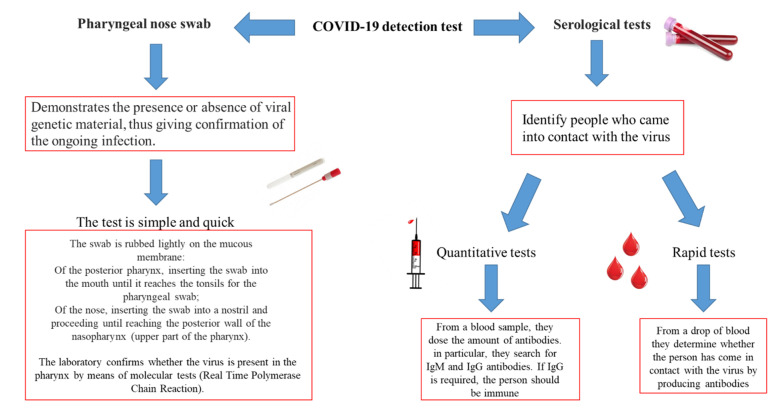
Diagnostic methods in use for the identification of COVID-19 infection.

To date, the methods used to identify COVID-19 are the nasopharyngeal swab and the serological test (Figure 6). The swab has greater sensitivity and specificity in recognition of COVID-19 compared to the serological test. The continuous studies of the medical-scientific community aim at the improvement of serological tests, which are rapid tests, which could be associated with common biochemical investigations in common use.

## 12. Current Therapies to Fight COVID-19 Disease

Currently, there is no proved therapy for the prevention or treatment of COVID-19. Immunotherapy (i.e., immunoglobulins and plasma therapy) has the potential to represent an efficient therapeutic option in patients with COVID-19. Immunotherapeutic protocols for COVID-19 include polyclonal antibody by plasma therapy, polypeptide hormone for the maturation of T cells, immunoglobulins, ACE2 immunoadhesin and a monoclonal antibody against the interleukin-6 [121]. COVID-19 patients receive these traditional therapies. The general treatments are very important to enhance the host immune response against RNA viral infection. Nutritional status of the host, lifestyle, physical exercise has not been considered, until recently, as a contributing factor to the emergence of viral infectious diseases. Indeed, it is well known that a healthy lifestyle with adequate nutrition is equivalent to a better and efficient immune defense [122]. These tend to have a better outcome and clinical development when they are affected by COVID-19. Now, there are several clinical trials to test different types of prevention therapy. Therefore, to improve the assurance of patients with COVID-19, further clinical trials and extensive randomized controlled studies are necessary to corroborate the efficacious role, safeness profile, and adverse results of all the test drugs [121]. However, any consent for the usage of new drug requests later clinical testing, followed by the approval of pervasive use by the pertinent regulatory body for medical treatment in every country. To date, the vaccine represents the most effective prevention drug [123].

Specifically, several vaccines have been developed (see Table 1). In particular, the vaccination campaign has begun throughout Europe starting from 27 December 2020. Hoping to permanently stop the spread of COVID-19.

## 13. Future Directions

The continuous appearance of new infected agents poses the need for an adaptation of the lifestyle both in daily life and in sport. In this regard, it would be advisable to draw up an international manifesto that would act as a guideline for both traditional and sports medicine. In fact, it would be necessary that in addition to the routine laboratory tests commonly performed, rapid tests were performed to identify aggressive pathogenic microorganisms such as COVID-19.

Therefore, this can be guaranteed by monitoring both athletes and ordinary citizens in order to safeguard both their health and that of those around them.

## 14. Conclusions

CoVs emerge periodically and present a rapid spread establishing serious infectious diseases. The universal vaccine is considered the maximum protection against the spread of the virus. At the same time, in order to prevent contagion, it is very important to adopt social distancing, the disinfection of common places, a suitable lifestyle that supports the immune system, personal hygiene. Therefore, in this scenario, the question of the scientific community arises whether sport can be a good enemy of the virus or an ally. The knowledge acquired to date through sports medicine and laboratory diagnostics suggests that moderate exercise can help the human body live better; on the other hand, intense exercise, if done inadequately, can cause numerous pathologies leading to a malfunction of the immune system. These events could therefore be the ideal stage for the onset of COVID-19 pathology.

## Figures and Tables

**Table 1:** Vaccines against COVID-19 currently in use.

Company	Type	Doses	How Effective	Storage	Cost for Doses
Oxford Uni-AstraZeneca	Viral vector 8 genetically modified virus)	X2	62–90%	4 °C	$4
Moderna	RNA (part of virus genetic code)	X2	95%	−20 °C up to 6 months	$33
Pfizer-BioNTech	RNA	X2	95%	−70 °C	$20
Gamaleya (Sputnik V)	Viral vector	X2	92%	4 °C	$10

## Data Availability

Not applicable.

## Abbreviations

CoVs	Coronaviruses
SARS-CoV	Severe acute respiratory syndrome—coronavirus
MERS-CoV	Middle East respiratory syndrome—coronavirus
COVID-19	Coronavirus disease 2019
URTI	Inflammation of the upper respiratory tract
AMP	Antimicrobial peptides
HNP1	Human alpha-defensin 1
HBD2	Human beta-defensin 2
HBD3	Human beta-defensin 3
HSV-1	Herpes virus simplex type I
HSV-2	Herpes virus simplex type II
CMV	Cytomegalovirus
AHSG	Human α-2-Heremans-Schmid Glycoprotein
IL-17	Interleukin-17
NF-kB	Nuclear factor kappa-light-chain-enhancer of activated B cells
IL-1	Interleukin-1
IL-6	Interleukin-6
IL-8	Interleukin-8
IL–21	Interleukin-21
IL–10	Interleukin-10
TNF–α	Tumor necrosis factor-alpha
TNF–β	Tumor necrosis factor-beta
ARDS	Acute respiratory distress syndrome
ACE2	Angiotensin-converting enzyme 2
TMPRSS2	Transmembrane Serine Protease 2
ACE1	Angiotensin-converting enzyme 1
TLR7/8	Toll-like receptors 7 and 8
